# How do digital platform capabilities affect service innovation in service manufacturing firms? The role of knowledge creation and environmental uncertainty

**DOI:** 10.1371/journal.pone.0333382

**Published:** 2025-09-26

**Authors:** Xuexin Liu, Lihua Jiang

**Affiliations:** College of Business Administration, Capital University of Economics and Business, Beijing, China; Universidade Europeia, Lisboa, PORTUGAL

## Abstract

The recent wave of scientific and technological revolutions, coupled with industrial advancements, has catalyzed the rise of service-oriented manufacturing enterprises in China. Despite their notable achievements, these enterprises grapple with a “service dilemma,” marked by a misalignment between input and output and ineffective service innovation. Digital platform capabilities emerge as a pivotal factor influencing service innovation. However, research has not explored their underlying mechanisms in depth. Therefore, this study investigates the impact of digital platform capabilities on service innovation within service-oriented manufacturing enterprises, examining the mediating role of knowledge creation and the moderating role of environmental uncertainty. Data were collected via structured questionnaires from middle and senior managers of service-oriented manufacturing enterprises, and hierarchical regression analysis was used to test the hypotheses. The findings reveal that digital platform capabilities significantly enhance service innovation. Moreover, knowledge creation mediates the relationship between digital platform capabilities and service innovation, while environmental uncertainty negatively moderates the link between knowledge creation and service innovation. Based on these insights, this paper proposes strategic recommendations to bolster service innovation by emphasizing digital platform capabilities, knowledge creation, and environmental uncertainty. These strategies aim to support the sustainable growth of service-oriented manufacturing enterprises in the rapidly evolving digital landscape.

## 1. Introduction

The recent scientific and technological revolution, coupled with the industrial revolution, has given rise to service-oriented manufacturing enterprises in China. These enterprises represent a significant transformation and upgrading of traditional manufacturing, integrating manufacturing and services deeply. Service innovation is crucial for these enterprises to gain a competitive advantage [1]. Unlike traditional manufacturing enterprises, service-oriented manufacturing enterprises emphasize customer participation in value co-creation and collaborate closely with upstream and downstream partners to create a product and service system tailored to personalized customer needs [2]. However, influenced by traditional value creation concepts, these enterprises have struggled to achieve genuine synergistic value creation, leading to suboptimal operations and ineffective service innovation [3]. This has resulted in a “service dilemma,” where investments in service innovation do not yield proportional returns. Addressing this dilemma and achieving effective service innovation is a critical challenge for these enterprises [4].

The advent and application of digital platforms, such as the industrial Internet, have significantly altered enterprise value creation models, providing new directions and impetus for overcoming the “service dilemma” [5]. Digital platforms help enterprises overcome information and knowledge search barriers, enabling quick and effective collection and integration of information from customers, partners, and competitors, thereby enriching resources for service innovation [6]. In 2021, David Soto Setzke believed that technology is a fundamental enabler of service innovation, especially in mature organizations [7].These platforms offer dynamic adjustment advantages through their flexible configuration [8], versatility, compatibility, and scalability. However, the information and knowledge from these platforms require active creativity from enterprise personnel, involving the repeated conversion of explicit and tacit knowledge to generate new concepts, knowledge, and services [9]. Thus, knowledge creation is vital for realizing service innovation through digital platform capabilities. Furthermore, the uncertainty in the external environment exacerbates the challenges of service innovation [10]. High levels of environmental uncertainty can delay knowledge creation, making it difficult to meet customer needs and achieve service innovation. Nevertheless, Nor’Aini Yusof argued that when a company is more innovative, its innovative behavior will lead to better business performance [11].

The current research on the consequences of digital platform capabilities can be categorized into two levels: organizational and social. At the organizational level, digital platform capabilities influence five key areas: organizational performance, innovation, network capabilities, dynamic capabilities, and international development. Specifically, regarding organizational performance and innovation, companies leverage digital platforms to share and optimize resource allocation [12]. Digital foundations with high openness can comprehensively enhance digital platforms, thereby improving organizational performance and innovation levels [13]. In terms of organizational network capabilities, robust digital platform capabilities offer data transmission and integration functions, enabling seamless connection between enterprises and their upstream and downstream partners, and thus strengthening organizational network capabilities [14]. Moreover, the variety of digital technologies such as big data and blockchain, and the richness of digital platforms, are positively correlated. The more diverse the technologies, the stronger the digital platform capabilities, and the higher the organizational network capabilities [15]. Regarding organizational dynamic capabilities, digital platform capabilities facilitate the integration of digital information and knowledge within enterprises. The integration of a large amount of effective information provides a basis for quick responses to critical decisions, thereby enhancing dynamic capabilities [16]. In terms of organizational international development, digital platform capabilities enable enterprises to fully sense market demands. Leveraging the high speed, low cost, and accessibility of the internet, they promote the global prevalence of digital e-commerce [17]. At the social level, digital platform capabilities significantly impact national development. Companies with digital platform capabilities can better utilize digital technologies to enhance the innovation ecosystem through multiple channels, collect digital information, and prepare for the digital transformation of enterprises nationwide [18]. Additionally, the digital platform capabilities of companies can influence societal digital development. By deeply linking upstream and downstream partners, digital industrial clusters are formed, further meeting market and user demands, improving the quality and quantity of digital products, and accelerating the digital transformation of the entire society [19]. The research on the influencing factors of service innovation mainly focuses on two aspects: internal and external factors. Internal factors primarily include corporate strategic management, innovation input, and human capital. Effective transformation of capabilities needed by manufacturing enterprises by managers is conducive to enhancing service innovation [20]. Increasing innovation input and effectively absorbing internal and external resources have a significant positive impact on service innovation [21]. The knowledge absorption ability of employees can significantly increase the indirect impact of customer participation in service innovation, thereby improving service innovation performance [22]. External factors mainly include tracks and actors. Tracks involve the spread of ideas or logic, covering areas such as technology, systems, and management; actors refer to customer groups that influence service innovation, including customers, suppliers, competitors, government departments, etc. Barrios et al. (2022) found through the SDL method that implicit ideas play an important role in shaping the service innovation ecosystem [23]. Peng and Li’s (2021) questionnaire survey shows that customer participation has a significant positive impact on service innovation [24]. Homayounfard and Zaefarian (2022) found in their research on the opportunities and challenges of service innovation that suppliers in B2B affect the process and outcomes of service innovation [25].

To sum up, while existing studies have thoroughly examined digital platform capabilities and service innovation, the following research gaps persist. First, most prior studies center on overall industries and fail to adequately account for manufacturing’s uniqueness. Manufacturing service innovation, which seeks to enhance product value-added, integrates manufacturing and services. Yet, manufacturers have relatively scarce resources and capabilities in service businesses, differing significantly from service firms. Second, few existing studies conduct a structural analysis of digital platform capabilities and explore their impact on service innovation. Most current research treats digital platform capabilities as a contextual factor in digital transformation, with limited direct examination of their effect on service innovation [26,27]. Additionally, when delving into service innovation drivers, there is insufficient in-depth exploration of firms’ digital platform capabilities. Third, regarding research on mechanisms, studies on how digital platform capabilities affect service innovation through knowledge creation mechanisms are inadequate. Given the widespread application of the Industrial Internet and other digital platforms in manufacturing, there is an urgent need to explore new ways to enhance service innovation performance through firms’ digital platform capabilities. Against this background, this paper addresses the following key research questions: (1) Do digital platform capabilities significantly affect service innovation in manufacturing firms? In terms of the composition of digital platform capabilities, do integration and analysis capabilities, as well as reconfiguration capabilities, have different effects on service innovation in manufacturing firms? (2) Does knowledge creation act as a mediator in the process of digital platform capabilities influencing service innovation in manufacturing? How does knowledge creation transform digital platform capabilities into service innovation outcomes? (3) Does environmental uncertainty moderate the relationship between digital platform capabilities and service innovation in manufacturing? How does this relationship change under varying levels of environmental uncertainty?

The marginal contributions of this paper are as follows: (1)This study broadens the research perspective on digital platform capabilities. Prior studies have mainly explored digital platform capabilities from a macro perspective, such as business model innovation. This study innovatively examines them from a micro viewpoint, breaking them down into two dimensions: “digital platform integration and analysis capabilities” and “digital platform reconfiguration capabilities”. In the context of manufacturing enterprises with integrated manufacturing and service, it verifies the differential effects of these two dimensions on service innovation. While digital platform integration and analysis capabilities drive knowledge accumulation through data processing, digital platform reconfiguration capabilities promote service model innovation through dynamic resource adjustment. This breakdown transcends the traditional single dimension framework, offering new insights and directions for related research. (2) This study expands the research dimensions of service innovation. Prior studies have mostly focused on traditional manufacturing or entire industries, treating digital platform capabilities as a background factor in digital transformation, and have not adequately explored the unique aspects of service-oriented manufacturing enterprises. This study concentrates on these enterprises and delves into how digital platform capabilities affect their service innovation. It creates a three – dimensional service innovation framework of “capabilities-knowledge-environment”. By integrating digital platform capabilities (with two dimensions), knowledge creation (as an intermediary mechanism), and environmental uncertainty (as a moderating variable), it forms a complete logical chain of “driver—transmitter—boundary”. This not only broadens the industry perspective of service innovation research but also, starting from the unique business models and value creation logic of service-oriented manufacturing enterprises, reveals how digital platform capabilities promote service innovation through unique paths such as integrating manufacturing and service resources and optimizing service interaction models. This in-depth exploration of the particularity of service innovation in service-oriented manufacturing enterprises introduces new dimensions and perspectives into service innovation research. (3)This study reveals the boundary mechanisms of service innovation. Previous research on environmental factors of service innovation often stays at the conceptual level. This study, from the perspective of “internal capabilities—external environment” interplay, explores the mechanism black box of service innovation in service-oriented manufacturing enterprises. It introduces environmental uncertainty as a moderator and shows its significant negative moderating effect on the knowledge creation-service innovation relationship. In high uncertainty environments with rapid technological iteration, insufficient timeliness of knowledge creation can cause service innovation lag. In contrast, in low uncertainty environments with stable demand, systematic knowledge accumulation can be transformed into innovation results. This finding extends environmental factor analysis from a binary “whether to influence” judgment to a continuous dimensional analysis of “how to influence”, offering enterprises more targeted guidance for promoting service innovation under different environmental conditions.

## 2. Theoretical analysis and research hypothesis

Grounded in an “external institutions–internal resources–intermediary processes–innovation outcomes” logic chain, this paper constructs an integrative, multi-theoretical framework that systematically elucidates how digital-platform capabilities shape service innovation in servitized manufacturing firms. Institutional theory provides the external-contextual anchor: formal and informal institutions simultaneously incentivize firms to cultivate digital-platform capabilities (i.e., integration-and-analysis and reconfiguration capabilities) via legitimacy- and mimicry-based mechanisms, yet may also amplify environmental uncertainty when institutional rules shift or conflict. Complementarily, the resource-based view supplies the internal-resource logic, conceptualizing digital-platform capabilities as heterogeneous resources that are valuable, rare, inimitable, and organizable—thereby constituting a central driver of service innovation. Knowledge-creation theory serves as the intermediary transformation bridge, positing that such capabilities must activate the “socialization–externalization–combination–internalization” spiral to convert external and internal information into new knowledge that fuels service innovation. Finally, environmental uncertainty—encompassing institutional, market, and technological dimensions—is introduced as a boundary condition expected to attenuate the knowledge-creation–service-innovation nexus. These theories interlock sequentially to yield the overarching logic: “institutional environment→digital-platform capabilities→knowledge-creation conversion→service innovation (moderated by environmental uncertainty).” The resultant framework furnishes a coherent theoretical foundation for subsequent hypothesis development and empirical examination.

### 2.1. Digital platform capabilities and service innovation for service-oriented manufacturing companies

#### 2.1.1. Digital platform capabilities.

Zhu (2015) first conceptualized platform capability as the ensemble of technological resources that platform firms deploy to support online digital activities [28]. Drawing on dynamic-capability theory, Jin and Hurd (2018) extended Zhu’s framework to the digital context and introduced the notion of “digital platform capability,” defined as a firm’s ability to enlarge market share through platform-based search [29]. This conceptualization aligns with the dynamic-capability emphasis on adaptive resource reconfiguration and caters to the real-time data integration and dynamic asset restructuring demanded by big-data and cloud-computing infrastructures. In contemporary industry settings, non-digital platforms—such as physical trade fairs or offline supplier alliances—rely on manual information exchange, exhibit limited scalability, and fail to synchronize resources in real time. Conversely, digital platforms (e.g., industrial-internet platforms and cloud-based collaboration systems) leverage digital technologies to integrate internal and external data, dynamically reconfigure resources, and facilitate inter-organizational coordination. These distinctive attributes render “digital platform capability” a context-specific construct that is qualitatively different from Zhu’s original “platform capability.” Although non-digital platforms still exist—particularly in regions with low digital penetration or in certain offline industries—they offer servitized manufacturing firms far less utility than digital platforms when the objective is data-driven service innovation. Blaschke et al(2018), from the perspective of platform ecosystem theory [30], defined it as the ability to establish zero-boundary customer connections via digital platforms, reflecting the theory’s focus on networked value creation. Recent research has extended digital platform capabilities to the circular economy. Nemilentseva et al.’s (2025) review highlights that digital platforms can achieve resource cycling and sustainable value creation in developing countries through integration and reconfiguration capabilities [31]. This addresses waste management and resource scarcity challenges and confirms that digital platform capabilities promote adaptive resource optimization beyond organizational boundaries, within broader ecological and socio-economic contexts.

Building on these theoretical foundations, this study anchors its definition in the dynamic capabilities framework, which emphasizes firms’ capacity to integrate, build, and reconfigure internal and external competences to address rapidly changing environments. Considering the research context, digital platform capability is defined as an enterprise’s ability to obtain effective internal and external information via digital platform channels, driving optimization and innovation centered on a “platform network.” These channels provide standards, connections, rules, and IT capabilities to coordinate digital production, search, and interaction in response to changing customer needs. Digital platform capabilities include digital platform integration and analysis capabilities and digital platform reconfiguration capabilities. The construct comprises two dimensions rooted in theoretical literature: Digital platform integration and analysis capabilities is consistent with the resource-based view, referring to an enterprise’s ability to collect, store, process, and analyze both internal and external data, which highlights the value of rare and inimitable data resources. Digital platform reconfiguration capabilities is consistent with the dynamic capabilities theory, referring to the ability to flexibly configure internal and external resources through platform architecture design, enabling continuous adaptation of digital technology systems [32].

Building on the institutional theory proposed by DiMaggio and Powell (1983), which emphasizes two core mechanisms—the coercive mechanism of formal institutions and the mimetic mechanism of informal institutions [33]. We argue that the digital platform capabilities of service-oriented manufacturing firms are not solely driven by internal resources. Instead, they are significantly shaped and incentivized by the external institutional environment. At the formal institutional level, local governments in China (e.g., in Jiangsu and Zhejiang provinces, the key research contexts of this study) have implemented targeted policies to promote digital transformation, such as direct subsidies for digital infrastructure construction and tax incentives for firms investing in industrial internet platforms [18,19]. These policies effectively reduce the financial barriers for firms to build digital platforms: subsidies cover a portion of the costs of developing data collection systems (a core component of integration and analysis capabilities), while tax breaks alleviate the pressure of investing in modular function design (a key aspect of reconfiguration capabilities). As a result, firms in these regions are more motivated and capable of enhancing both dimensions of digital platform capabilities. At the informal institutional level, industry associations have played a pivotal role in establishing “digital platform standards,” such as unified data interface specifications and cross-enterprise data sharing protocols. These standards gradually evolve into industry norms, and firms face pressure to conform to avoid “institutional illegitimacy”—a status that could undermine their partnerships with upstream/downstream stakeholders and customer trust [33,34]. To gain legitimacy, many service-oriented manufacturing firms mimic the platform architectures and functional configurations of leading enterprises in the industry. This mimetic behavior accelerates the maturity of their own digital platform reconfiguration capabilities, as they can leverage proven designs to avoid trial-and-error costs.

In summary, the development of digital platform capabilities in service-oriented manufacturing firms is a hybrid outcome of internal resource allocation and external institutional push. This institutional perspective not only enriches our understanding of the antecedents of digital platform capabilities but also reinforces the necessity of exploring how such capabilities drive service innovation—especially in the context of China’s distinctive institutional environment for digital transformation.

#### 2.1.2. Service innovation.

In 1988, Vandermerwe and Rada. proposed the concept of servitization, suggesting it is a shift for manufacturing firms from merely product provision to offering a “product - service package” centered on client firms, and a process of establishing this package as a source of value-addition [35]. Bian and Fan (2024) noted that service-oriented manufacturing enterprises are a new enterprise form emerging from the digital transformation of manufacturing [36]. These firms seamlessly integrate manufacturing and service activities, leveraging digital technologies to enhance their position in the global value chain. Building on prior studies, this paper views servitization as a new industrial form integrating manufacturing and services. Service-oriented manufacturing enterprises are those that innovate and optimize production organization and operational management, increase the proportion of service elements in inputs and outputs, and thus transform from primarily processing and assembly to a “manufacturing + service” model.

Service innovation, a key concept in servitization, has been extensively studied. In 1987, Betz introduced the concept of service innovation, emphasizing the application of new technologies to original processes and technologies to improve service efficiency [37]. With changing consumer demands, Gallouj and Weinstein(1997) defined service innovation as product or service improvements made by enterprises to meet customer needs at different stages [38]. In 2015, Skalen et al. described service innovation as a way for enterprises to seek or utilize external resources through technological means, integrating or creatively providing new service values [39]. Drawing upon these seminal studies, this research defines service innovation as an activity undertaken by servitized manufacturing firms. These firms utilize digital platforms, leveraging the exchange value of products as a foundation. Based on this foundation, they create value by offering users customized solutions that are tailored to their specific needs. In specific terms, firms make use of digital technologies, cloud computing, big data, and other such tools. They employ these tools to build or integrate intelligent digital platforms and deeply explore external resources, transforming them into actual productive capacity. By redesigning service interaction models, firms can achieve more efficient and personalized communication with users; optimizing service processes can eliminate redundant steps and enhance service response times; and innovating product functions can provide users with unexpected experiences. Whether introducing novel service designs, creating pioneering user interaction scenarios, building unique service delivery systems, or iterating and upgrading existing services, the ultimate goal is always to comprehensively enhance service quality and efficiency. In doing so, firms can increase user satisfaction and establish a unique competitive advantage in the face of intense market competition.

#### 2.1.3. Digital platform capabilities and service innovation.

Institutional theory provides a critical foundation for the resource-based view. The resource-based view posits that the firm is a bundle of unique resources and capabilities that constitute the primary source of competitive advantage [40,41]. The institutional environment, however, largely determines which resources firms can acquire, accumulate, and deploy. Industrial policies may channel firms toward specific technologies or talent pools, while industry norms shape how resources are integrated and managed. For servitized manufacturers, the prevailing institutional climate surrounding digitalization compels them to build digital platforms; consequently, digital platform capabilities have evolved into a distinctive and valuable firm resource.Traditional RBV attributes competitive advantage to heterogeneous resources and capabilities [42], yet it offers limited insight into how those resources emerge and evolve [43]. Applying the VRIO lens to servitized manufacturers reveals why digital-platform capabilities constitute such a resource.Value. Superior integration-and-analytics modules mine massive internal and external data to detect latent demand and fine-tune service offerings, while reconfiguration routines allow rapid, low-cost adjustments to processes and resource mixes, neutralizing threats and exploiting market opportunities. Rarity. Building a fully fledged digital platform demands heavy, sustained investment in capital, technology, and specialist labour; only a minority of firms can assemble the requisite bundles. Imitability. Each platform embeds not only complex code and architecture but also firm-specific business processes, governance practices, and longitudinal customer data that are causally ambiguous and socially complex, making full replication prohibitively costly and time-consuming. Organization. Effective administrative structures and coordination mechanisms ensure that platform capabilities are systematically leveraged and continuously renewed rather than lying idle or depreciating. Dynamic-resource theory further argues that in volatile environments sustainable advantage requires continual integration, analysis, and reconfiguration of resources [44]. Because services are intangible and heterogeneous, servitized manufacturers must embed innovation across the entire “manufacturing + service” flow; platform-centric firms use real-time data to orchestrate production, search, and interaction, enabling rapid response to shifting customer needs [17,21]. We therefore examine service innovation through the two-dimensional lens of digital-platform integration-and-analysis capability and digital-platform reconfiguration capability.

Digital platform integration and analysis capabilities encompass the overall process of collecting, analyzing, using, and interpreting data to transform it into actionable insights. This process creates value and builds competitive advantages for firms in today’s economy [45,46]. Drucker highlighted that in the new economy, a firm’s innovation capacity hinges on its ability to efficiently access external knowledge. In the digital economy, digital knowledge has become an indispensable resource for service-oriented manufacturing enterprises. However, merely collecting and analyzing internal data is no longer sufficient for enterprise development. Companies must mine, integrate, analyze, and utilize complex external data to identify information that truly suits their development strategies and promotes innovation.

Moreover, transforming data into actionable decisions requires not only efficient data processing techniques but also the ability to foster cross-departmental collaboration. Breaking down silos within organizations allows data to flow freely, unlocking its full potential and driving innovation. Lehrer et al. (2018) found that firms leveraging digital platforms to collect and analyze customer data exhibit stronger innovation capabilities, enabling them to offer customized products and services [47].

Service innovation theory emphasizes that innovation originates both internally and externally within a firm. Internally, enhancing digital platform integration and analysis capabilities allows service-oriented manufacturing enterprises to improve employees’ familiarity with business processes and supply chain dynamics. This familiarity fosters valuable creativity and effective innovation [48]. Externally, these capabilities enable firms to establish connections with external organizations, expand customer information databases, and accurately predict market trends. By providing personalized customer experiences, firms can reduce market risks and enhance the effectiveness of their service innovations.

In the external realm, digital platform integration and analysis capabilities play an even more pivotal role. By connecting with external organizations, enterprises can significantly expand their customer information databases, enabling precise market predictions. offering highly personalized products and services. This interaction also helps customers gain a deeper understanding of the enterprise’s product positioning and service culture, fostering long-term relationships. The synergy between internal and external processes reduces market risks and amplifies the effectiveness of service innovations, providing enterprises with a competitive edge in the market. Based on this analysis, we propose the following hypothesis:

H1a: The ability to integrate and analyze digital platforms in service-oriented manufacturing firms has a positive impact on service innovation.

Digital platform reconfiguration capabilities refer to a firm’s ability to utilize the flexible architecture of digital platforms to reconfigure internal and external resources [49]. This ability enables enterprises to proactively repair and optimize existing value-creating processes. It allows firms to overcome cognitive and operational barriers, bridge capability gaps with competitors, and achieve sustained optimization in dynamic environments.

Firstly, Value co-creation theory emphasizes that value creation is based on service exchanges between parties. During these exchanges, in-depth interactions and complementary strengths drive innovation. Service-oriented manufacturing enterprises with digital platform reconfiguration capabilities can leverage the modular architecture of digital platforms [50]. They can establish digital business models with other platform enterprises, exchange goods and services with stakeholders, share information, and collaborate within the platform ecosystem. This enables the rapid adaptation to new partners and applications, optimizing or replacing existing processes.

Additionally, digital platforms’ openness and transparency provide enterprises with valuable insights for developing new products or processes. drawing on established models. Firms can easily access customer feedback and preferences, enhancing the competitiveness of their new services [21]. This accelerates innovation in service-oriented manufacturing enterprises. Kopalle et al.(2020) explored how modular digital platforms offer effective pathways for resource reconfiguration, supporting enterprises in delivering high-quality, diverse innovative products and services [51].

In the digital economy, firms are not isolated but are part of a dynamic digital ecosystem. The openness of digital platforms attracts numerous developers, suppliers, and customers, forming an interdependent and co-evolving ecosystem. Within this ecosystem, enterprises can harness external innovation resources, collaborate on R&D projects, and share technological achievements with partners. This accelerates innovation iteration and forms a powerful innovative synergy, driving technological progress and industrial upgrading across the sector.

The stronger a service-oriented manufacturing enterprise’s digital platform reconfiguration capabilities, the richer its customer-related innovative elements, and the more likely it is to achieve disruptive innovation. Based on this, we propose:

H1b: The ability to reconfigure digital platforms in service manufacturing firms has a positive impact on service innovation.

### 2.2. The mediating role of knowledge creation

Knowledge creation, first proposed by Nonaka and Takeuchi in 1995, is a spiral process involving multiple aspects of individuals, teams, and organizations [52]. Similarly, Krogh (1998) sees knowledge creation as a process of intellectual activity that generates explicit and tacit knowledge through solidarity and collaboration among people from different backgrounds [53]. According to knowledge creation theory, the ability to utilize and generate new information and knowledge provides firms with a sustainable competitive advantage, enhances their ability to achieve strategic goals, and improves service innovation, including the development of innovative products and improved processes [54].

From an institutional-theory perspective, knowledge creation is not an isolated intra-organizational activity but is embedded in, and shaped by, the external institutional environment. Through the legitimacy mechanism, institutions provide both directional cues and resource support for knowledge creation, thereby functioning as a critical link between knowledge-creation theory and the resource-based view. RBV argues that heterogeneous resources are the source of sustained competitive advantage [43]; institutionally anchored knowledge creation serves as the pivotal bridge that converts “digital-platform resources” into “service-innovation advantage.” Specifically, after servitized manufacturers acquire customer-demand data, partners’ technological know-how, and competitors’ market feedback via the integration-and-analysis functions of their digital platforms, they must transform these fragmented pieces of information into actionable knowledge. Institutionalized knowledge frameworks—such as government-mandated data-classification standards—facilitate the screening, integration, and interpretation of dispersed data, allowing firms to identify latent customer needs with greater precision (e.g., detecting demand for eco-friendly customized services by aligning with policy-supported “green service” initiatives). Concurrently, firms can leverage partners’ innovative resources (e.g., technical solutions shared on industry knowledge platforms) to refine product and technology configurations, ultimately improving service models and elevating service-innovation performance [55].

Meanwhile, the compatibility and scalability of digital-platform reconfiguration capabilities, amplified by an enabling institutional environment, further intensify the efficacy of knowledge creation. On the one hand, platform compatibility allows firms to rapidly connect to institutionally endorsed external knowledge interfaces (such as government-sponsored university–industry–research knowledge-matching portals), thereby integrating heterogeneous knowledge inputs (e.g., university R&D findings and industry-association market forecasts) and broadening the scope of knowledge creation. On the other hand, platform scalability permits firms to dynamically adjust knowledge-storage and conversion modules in line with institutional cues; for instance, when policies promote “flexible services,” enterprises can swiftly add a “tacit-customer-needs-mining” module that accelerates the externalization of implicit knowledge (e.g., customers’ usage habits) into explicit service schemes, shortening the knowledge-creation cycle [56]. Institutionally empowered in this way, knowledge creation drives service innovation from both intra- and inter-organizational angles: internally, standardized knowledge training grounded in industry conventions upgrades employees’ service mind-sets and efficiency, while externally, institutionally legitimated technical knowledge—such as government-certified green-service technologies—fuels technology development, process re-engineering, and product design. The result is a coherent “institutional guidance→knowledge creation→service innovation” logic chain. Based on this analysis, the following hypotheses are proposed:

H2a: Knowledge creation mediates the relationship between digital platform integration and analysis capabilities and service innovation in service-oriented manufacturing firms.

H2b: Knowledge creation mediates the relationship between digital platform reconfiguration capabilities and service innovation in service-oriented manufacturing firms.

### 2.3. The moderating role of environmental uncertainty

The concept of uncertainty was first introduced by Knight in 1921, referring to the lack of complete or timely information about the development of events, which prevents accurate judgments. Environmental uncertainty arises when people cannot fully comprehend the development of events due to changes in the environment. Duncan (1972) initially elaborated on the impact of environmental uncertainty from a strategic management perspective, proposing a three-phase approach [57]. From an economic viewpoint, Priem et al (2002) define environmental uncertainty as the unpredictability that increases managers’ uncertainty about future predictions, thus raising risks for decision-makers [58].

It should be noted that sources of environmental uncertainty are not confined to conventional dimensions such as demand volatility or technological discontinuity; they also encompass institutional uncertainty—a dimension that is especially salient in transition economies and represents the core intersection of institutional theory and environmental uncertainty research. From an institutional perspective, uncertainty arises at two interrelated levels. First, the frequent revision of formal institutions—for example, recurrent amendments to government digital-transformation policies and the growing ambiguity of industry-specific regulations—makes it difficult for servitized manufacturers to formulate stable plans for digital-platform development and knowledge-creation trajectories [18,34]. Second, informal institutions are often characterized by normative ambiguity and conflict: industry associations have yet to establish universally accepted “digital-platform knowledge-sharing protocols,” while leading firms promote disparate technical standards and service routines. Consequently, firms confront a legitimacy-based choice dilemma in inter-organizational knowledge collaboration: adopting Firm A’s knowledge-management template may violate Firm B’s established conventions, and vice versa. Such informal institutional conflicts further intensify perceived environmental uncertainty [19,33].

Institutional uncertainty, when superimposed upon market- and technology-related turbulence, amplifies the overall magnitude of environmental uncertainty confronting servitized manufacturing firms. Corporate strategy scholars argue that environmental uncertainty slows down the rate of service innovation in firms [59]. High environmental uncertainty leads to rapid and complex changes in customer consumption preferences, partner resource acquisition, competitor innovation modes, and non-market events, limiting the time available for enterprises to create knowledge. This increased difficulty in integrating, analyzing, and reconfiguring information and knowledge further affects the service innovation of service-oriented manufacturing enterprises. In such uncertain environments, predicting consumer preferences accurately becomes challenging, making it difficult for enterprises to capture real market demand. Consequently, service innovation must evolve through continuous trial-and-error iterations [60].

Despite acquiring substantial external information and knowledge through digital platform integration, analysis, and reconfiguration capabilities, enterprises experience a lag in internal adjustment and digestion. The uncertain environment exacerbates the cost of coordinating resources for service innovation, reduces resource allocation efficiency, interferes with strategic decision-making, and slows down the process of service innovation. Conversely, when the external environment is more certain, enterprises have more time to screen, integrate, and experiment with information and knowledge, fostering successful service innovation activities with partners. Therefore, in highly complex and dynamic environments, the impact of knowledge creation on service innovation is weakened. Based on this analysis, the following hypothesis is proposed:

H3: Environmental uncertainty has a negative moderating effect on knowledge creation and service innovation.

**Fig 1:**
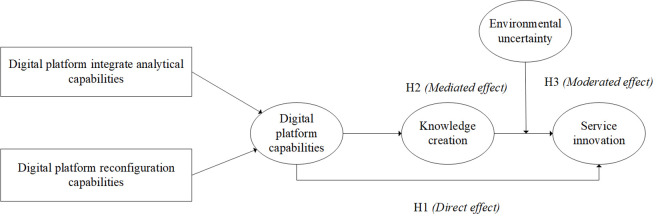
Theoretical model.

In summary, the theoretical model of this study is shown in Fig 1.

The model 1 shows the interrelationship between digital platform capabilities, knowledge creation, environmental uncertainty and service innovation in the context of the digital age. Digital platform capabilities include digital technology infrastructure, integration of platform functions, and data analysis capabilities. As a core factor, digital platform capability directly affects the service innovation ability of service-oriented manufacturing enterprises. This study aims to explore how digital platform capabilities can drive service innovation, especially through the mediating role of knowledge creation mechanisms. Knowledge creation includes knowledge accumulation, innovation ability, and technical exchange within the organization. Knowledge creation is a catalyst for service innovation, helping businesses generate new ideas and solutions. Knowledge creation mediates the gap between digital platform capabilities and service innovation, helping to translate the potential of digital platforms into actual innovations. Environmental uncertainties include factors such as technological changes, fluctuations in market demand, and policy adjustments. Environmental uncertainty increases the challenge of innovation for service-oriented manufacturing companies. Environmental uncertainty is considered to negatively moderate the relationship between knowledge creation and service innovation, that is, in a highly uncertain environment, the role of knowledge creation in promoting service innovation may be weakened. Service innovation includes the development of new services, the optimization of existing services, and the improvement of service processes and service quality. Service innovation is the key for service-oriented manufacturing enterprises to stand out from the competition. Service innovation is the ultimate goal, and the study aims to explore how to promote service innovation through digital platform capabilities and knowledge creation, while considering the impact of environmental uncertainty. In the model, digital platform capabilities are the main force driving knowledge creation, and knowledge creation is the key mechanism to promote service innovation. Environmental uncertainty may weaken this relationship and affect the effectiveness of enterprise service innovation.

## 3. Methods

### 3.1. Sample and data collection

The study collected data between April 1, 2024, and June 30, 2024, focusing on service-oriented manufacturing enterprises in Jiangsu and Zhejiang provinces. The selection of these regions was based on three main considerations: First, these provinces are representative of advanced manufacturing in China. They are key demonstration zones for manufacturing digital transformation, with numerous servitized enterprises that actively integrate digital technologies into their operations, offering rich scenarios for studying digital platform capabilities and service innovation. Second, they provide theoretical validation value. There are significant differences in the digital maturity of enterprises within these provinces, from basic informatization to full-chain intelligence. This allows for capturing the varying impacts of different levels of digital platform capabilities on service innovation, addressing the “context - specific” gap in existing research. Third, there is a good fit with industrial policies. These provinces have implemented the “Action Plan for the Transformation of Manufacturing to Servitization,” and the policy orientation aligns with the practice – oriented nature of this study. The research findings can directly inform local industry upgrades. Questionnaires were distributed both online and offline, targeting middle and senior management personnel who have a deep understanding of the enterprise’s digital platform capabilities, service innovation initiatives, and overall strategic direction. A total of 480 questionnaires were distributed, with 408 valid responses received, representing a valid response rate of 85%.

Before the formal survey, a pre-survey was conducted among servitized manufacturing firms using digital platforms. We distributed 52 questionnaires and received 47 valid responses, which we analyzed for reliability and validity to refine the survey content. Additionally, in-depth interviews with executives from several companies provided qualitative insights into the research topic, further enhancing the questionnaire’s relevance and validity. The interview feedback helped clarify the research questions and ensured the questionnaire items effectively captured valuable constructs.

In order to mitigate potential respondent bias, the following measures were taken in this study: Firstly, a stratified random sampling technique was applied in selecting the samples to ensure that firms of different sizes, sectors, and levels of digital transformation were included. Secondly, the anonymity of the participants was strictly maintained. No personal identifiers were collected in the questionnaires, data storage was encrypted, and participants were encouraged to provide genuine and objective responses. Thirdly, the purpose and significance of the study were communicated transparently to the participants to enhance their engagement and the quality of their responses.

Valid questionnaires included responses from: 36% of the company’s top management and middle and senior management of the R&D department; 36% of personnel in corporate strategy development and core product development; 46% of middle and senior management of the marketing department; and the middle and senior management of the sales department. Additionally, 60% of the sample had more than 8 years of work experience, and 19% had 5–8 years of experience. Companies established for 5–10 years and those with over 10 years constituted 90% of the total sample. Companies with 101–500 employees accounted for 60% of the sample. Regarding company type, 77% were private companies and 10% were state-owned.

### 3.2. Ethical consideration

This study did not obtain ethical approval for the following reasons: This study aims to invite middle and senior managers of service-oriented manufacturing companies with a digital background to complete questionnaires and investigate the operating conditions of their companies. Firstly, voluntary informed consent forms signed by the research participants were obtained prior to the commencement of the research. The relevant content and purpose of the study were well within the scope of standardized informed consent. Secondly, this study utilized anonymized information data for research purposes. Thirdly, this research posed no harm to the human body and did not involve sensitive personal information or commercial interests. Moreover, this study adhered to the principles expressed in the Declaration of Helsinki. Additionally, this research met the ethical exemption requirements stipulated in the “Ethical Review Measures for Life Sciences and Medical Research Involving Humans” promulgated by China, and thus was exempted from ethical review.

### 3.3. Measurement of variables

The variable measurements include digital platform integration and analysis ability, digital platform reconfiguration ability, knowledge creation, environmental uncertainty, and service innovation. To ensure the validity of the scales, mature scales that have been used domestically and internationally are adopted. The scales are evaluated using a Likert-5-point scale, where 1 indicates strong non-conformity, 2 indicates comparative non-conformity, 3 indicates uncertainty, 4 indicates comparative conformity, and 5 indicates strong conformity.

**Digital Platform Capability.** Referring to the measurement scales developed by Cenamor et al. (2019) and Zhu et al.(2015) to assess digital platform capabilities, the digital platform capabilities are divided into digital platform integration and analysis capability and digital platform reconfiguration capability, each measured with four items [14,28]. For digital platform integration and analysis capability [14], items include “Enterprises can easily access the systems of platform partners to collect, transfer, and integrate platform data information.” For digital platform reconfiguration capability, an example item is “The digital platform enables us to utilize existing functional modules to configure new products.” The reliability of these scales are 0.78 and 0.88, respectively.

**Service Innovation.** Referring to the Service Innovation Scale developed by Carmona-Lavado, service innovation is understood as a product or service that provides uniqueness and value to a firm’s customers, meeting market demands innovatively [61]. The scale consists of six items, such as “The service provided by the firm is unique or innovative to the customer.” The reliability of this scale is 0.88.

**Knowledge Creation.** Utilizing the Knowledge Creation Measurement Scale developed by Zheng et al (2011), this scale covers four aspects: internal management information knowledge, market information knowledge, technical information knowledge, and product information knowledge [62]. It consists of five items, including “The enterprise conceives new ideas or develops new solutions to problems according to changes in the external environment to update management experience and skills.” The reliability of this scale is 0.83.

**Environmental Uncertainty.** This is measured using the scale developed by Miller and Friesen (1983), which has demonstrated good reliability and validity [63]. The scale includes six items, such as “Customers of the company are constantly asking for new products and services.” The reliability of this scale is 0.85.

**Control Variables:** To avoid the influence of other potential variables on the research results, the following variables were included in the analysis, which were selected a priori based on theoretical foundations. First, according to the study by Abou-Foul et al (2021), the older an enterprise is, the higher its market participation and the richer its service innovation experience [64]. Enterprises with a longer history possess more mature organizational processes and resource accumulation, which affect their level of leveraging digital platform capabilities. Therefore, enterprise age was considered as a control variable. Second, based on the research by Govindarajan et al (2011), enterprise size influences the speed and scale of digital platform implementation [65]. Larger enterprises typically have more abundant resources and stronger R&D capabilities, but may also face higher management complexity. Thus, enterprise size was considered as a control variable. Then, according to Zhang and Zhu (2021), the ownership structure of an enterprise affects its willingness to invest in digital transformation and service innovation [66]. State-owned enterprises and non-state-owned enterprises differ in corporate strategies and resource acquisition channels, leading to differences in their attitudes toward accepting digital transformation. Therefore, enterprise ownership was considered as a control variable. Finally, based on the study by Wang et al (2022), enterprise cooperation experience can enhance the ability to achieve value co-creation through digital platforms [26]. Enterprises with richer cooperation experience often have more complete network capabilities and knowledge integration capabilities. Hence, enterprise cooperation experience was considered as a control variable.

### 3.4. Statistical analysis

This study used SPSS 26.0 for statistical testing. First, confirmatory factor analysis (CFA) was performed on the collected variables, and Harman’s single-factor test was employed to address potential common method bias issues. Second, descriptive statistics and correlation analysis were conducted on the variables to preliminarily validate the hypotheses. Finally, hierarchical regression and bootstrap analysis were used to test the main effects, mediating effects, and moderated mediating effects of the model.

The choice of hierarchical regression analysis over structural equation modeling (SEM) in this study is primarily due to the need to clearly distinguish the incremental contributions of main effects, mediating effects, moderating effects, and control variables. Hierarchical regression allows for the stepwise introduction of variables to intuitively demonstrate the change in explanatory power of each layer of the model, providing more direct statistical evidence through coefficient significance tests. In contrast, SEM typically evaluates the overall model fit at once, and global fit indices may obscure local relationships.

## 4. Results

### 4.1. Validation factor analysis

In this study, the discriminant validity of the scales was evaluated through the calculated Average Variance Extracted (AVE) values, and the convergent validity of the questionnaire scales was assessed through the Composite Reliability (CR) values, as shown in Table 1.

**Table 1:** Results of validated factor analysis (N = 408).

Variables	Items	Factor loading	*AVE*	*CR*
Digital platforms integrate analytical capabilities	DI1	0.732	0.538	0.823
	DI2	0.769		
	DI3	0.674		
	DI4	0.754		
Digital platform reconfiguration capabilities	DR1	0.819	0.731	0.915
	DR2	0.930		
	DR3	0.847		
	DR4	0.818		
Service Innovation	SI1	0.770	0.591	0.900
	SI2	0.777		
	SI3	0.759		
	SI4	0.793		
	SI5	0.764		
	SI6	0.749		
Knowledge creation	KC1	0.768	0.545	0.857
	KC2	0.706		
	KC3	0.716		
	KC4	0.753		
	KC5	0.747		
Environmental uncertainty	EU1	0.725	0.542	0.877
	EU2	0.715		
	EU3	0.774		
	EU4	0.754		
	EU5	0.733		
	EU6	0.715		

The lowest factor loading for the digital platform integration and analysis capability scale is 0.674, higher than the critical value of 0.5. The AVE value is 0.538 and the CR value is 0.823, indicating good convergent validity. The lowest factor loading for the digital platform reconfiguration scale is 0.818, the AVE value is 0.731, and the CR value is 0.915, all exceeding the critical values, confirming good convergent validity. The lowest factor loading for the Service Innovation Scale is 0.749, the AVE value is 0.591, and the CR value is 0.900, suggesting strong convergent validity. The lowest factor loading for the Knowledge Creation Scale is 0.706, the AVE value is 0.545, and the CR value is 0.857, indicating good convergent validity. The lowest factor loading for the Environmental Uncertainty Scale is 0.715, the AVE value is 0.542, and the CR value is 0.877, demonstrating strong convergent validity.

### 4.2. Common method bias test

To address potential common method bias, which can occur due to subjective factors influencing the employees’ responses, an unrotated principal component analysis of the question items was performed using SPSS 26.0. The results show that the total variance explained by the first factor is 20.298%, which is significantly lower than half of the cumulative explanation of the other factors (79.702%). This indicates that there is no significant common methodological bias in the questionnaire data, as detailed in Table 2.

**Table 2:** Results of unrotated principal component analysis.

Variables	Initial eigenvalue			Extract the sum of the squares of the loads		
	Total	Percentage of variance	Cumulative %	Total	Percentage of variance	Cumulative %
1	17.253	20.298	20.298	17.253	20.298	20.298
2	11.405	13.417	33.715	11.405	13.417	33.715
3	11.043	12.992	46.708	11.043	12.992	46.708
4	10.845	12.759	59.466	10.845	12.759	59.466
5	10.180	11.976	71.442	10.180	11.976	71.442
6	5.232	6.155	77.597	5.232	6.155	77.597
7	4.703	5.532	83.129	4.703	5.532	83.129
8	3.131	3.683	86.813	3.131	3.683	86.813
9	1.014	1.193	88.006	1.014	1.193	88.006

### 4.3. Descriptive statistics and correlation analysis

According to the results in Table 3, the mean and standard deviation of each variable align with expectations. The correlation coefficients between each variable are all below 0.7, indicating no covariance issues among the variables. Digital platform integration and analysis capability is significantly and positively correlated with knowledge creation (r = 0.374, p < 0.01), environmental uncertainty (r = 0.319, p < 0.01), and service innovation (r = 0.364, p < 0.01). Similarly, digital platform reconfiguration capability is significantly and positively correlated with knowledge creation (r = 0.253, p < 0.01), environmental uncertainty (r = 0.275, p < 0.01), and service innovation (r = 0.278, p < 0.01). There is also a significant positive correlation between knowledge creation and service innovation (r = 0.313, p < 0.01).

**Table 3:** Descriptive statistical analysis and correlation coefficient matrix (N = 408).

Variables					*Mean*		*SD*	1		2	3		4
1.Company age					3.31		0.747	1					
2.Company size					2.26		0.817	0.216**		1			
3.Company characteristic					2.12		0.695	−0.198**		0.006	1		
4.The company’s experience with other companies					3.75		1.146	0.154**		−0.034	−0.083		1
**Variables**	***Mean***	***SD***	**1**	**2**	**3**	**4**	**5**		**6**	**7**		**8**	**9**
5.Digital platforms integrate analytical capability	15.46	3.060	0.247**	0.243**	−0.033	0.139**	1						
6.Digital platform reconfiguration capability	14.52	4.034	0.099*	0.106*	−0.010	−0.014	0.321**		1				
7.Service Innovation	22.22	5.157	0.045	0.095	−0.019	0.108*	0.364**		0.278**	1			
8.Knowledge creation	20.34	3.713	0.035	0.057	−0.007	0.049	0.374**		0.253**	0.313**		1	
9.Environmental uncertainty	23.99	4.426	0.062	0.100*	−0.057	0.038	0.319**		0.275**	0.269**		0.310**	1

Notes: **P<0.01

### 4.4. Hypothesis testing

#### 4.4.1. Main effect and mediation effect tests.

This study employs hierarchical regression analysis to test the hypotheses, with the results presented in Table 4. Model 2 shows that digital platform integration and analysis capability has a significant positive impact on service innovation (β = 0.514, p < 0.01), supporting Hypothesis H1a. Similarly, digital platform reconfiguration capability significantly positively impacts service innovation (β = 0.273, p < 0.01), verifying Hypothesis H1b.

**Table 4:** Results of regression analysis of main and mediating effects.

Variables	Service innovation					Knowledge creation	
	Model1	Model 2	Model 3	Model 4	Model 5	Model 6	Model 7
Company age	0.034	−0.468	0.003	−0.353	−0.115	0.075	−0.310
Company size	0.615	0.114	0.509	0.173	0.411	0.251	−0.134
Company characteristic	−0.069	−0.105	−0.068	−0.097	−0.075	−0.001	−0.030
The company’s experience with other companies	0.495*	0.353	0.429*	0.300	0.463***	0.157	0.036
Digital platforms integrate analytical capability		0.514***		0.478***			0.420***
Digital platform reconfiguration capability		0.273***			0.269***		0.139***
Knowledge creation			0.422***	0.283***	0.350***		
$R^{2}$	0.022	0.170	0.113	0.175	0.154	0.164	0.164
F	2.218	13.680***	10.264***	14.155***	12.157***	13.128***	13.128***

In Model 3, after controlling for other variables, knowledge creation significantly positively influences service innovation (β = 0.383, p < 0.01). In Model 4, adding knowledge creation to the established relationship between digital platform integration and analysis capability and service innovation shows that knowledge creation continues to have a significant positive effect on service innovation (β = 0.317, p < 0.01), and the regression coefficient of digital platform integration and analysis capability decreases from 0.514 to 0.478. This indicates that knowledge creation partially mediates the relationship between digital platform integration and analysis capability and service innovation, verifying Hypothesis 2a. Model 5 shows that when knowledge creation is added to the relationship between digital platform reconfiguration capability and service innovation, knowledge creation still significantly positively affects service innovation (β = 0.312, p < 0.01). The regression coefficient of digital platform reconfiguration capability decreases from 0.273 to 0.269, indicating that knowledge creation partially mediates this relationship as well, supporting Hypothesis 2b.

To further test the mediating effect of knowledge creation, a Bootstrap test was conducted on Model 4 using the Process plug-in. The results presented in Table 5. The results indicate that the indirect effect of digital platform integration and analysis capability on service innovation through knowledge creation is 0.135, with a 95% confidence interval of [0.056, 0.226], supporting Hypothesis 2a. The indirect impact of digital platform reconfiguration capabilities on service innovation through knowledge creation is 0.081, with a 95% confidence interval of [0.029, 0.033], further supporting Hypothesis 2b.

**Table 5:** Mediating effect test results.

Route	Type of effect	Efficiency value	Standard error	95% confidence interval
Digital platforms integrate analytical capabilities→Knowledge creation→Service innovation	direct effect	0.478	0.087	[0.360, 0.650]
	indirect effect	0.135	0.044	[0.056, 0.226]
Digital platform reconfiguration capability→Knowledge creation→Service innovation	direct effect	0.269	0.061	[0.148, 0.389]
	indirect effect	0.081	0.029	[0.033, 0.046]

#### 4.4.2. Moderating effect test.

The moderating role of environmental uncertainty was tested using the Process program developed by Hayes(2012), with the results shown in Table 6 [67]. The interaction term between knowledge creation and service innovation has coefficients (β = −0.216, p < 0.01) indicating that environmental uncertainty negatively moderates the relationship between knowledge creation and service innovation, verifying Hypothesis 3.

**Table 6:** Tests of the moderating effect of environmental uncertainty.

Norm	*Coeff*	*R* ^*2*^	*F*	*df1*	*df2*	*P*
Knowledge creation× Environmental uncertainty	−0.050	0.030	15.479	1	400	0.0001
Grouping of moderating variables	Effect	SE	t	p	LLCI	ULCI
Low regulation group	0.608	0.081	7.545	0.000	0.450	0.767
Central regulation group	0.387	0.060	0.459	0.000	0.269	0.505
High regulation group	0.165	0.084	1.974	0.049	0.001	0.330

**Fig 2:**
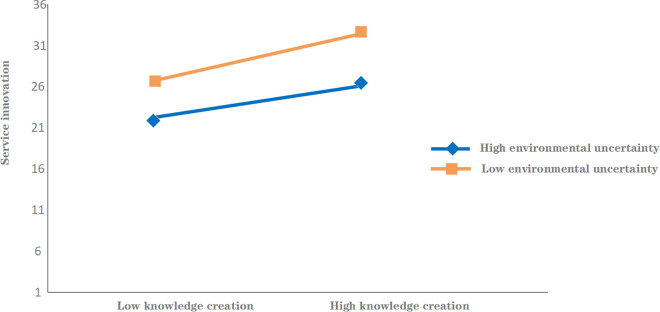
The moderating effect of environmental uncertainty.

To visually demonstrate the moderating effect, environmental uncertainty was plotted to show its moderating influence on the relationship between knowledge creation and service innovation. As depicted in Fig 2, high environmental uncertainty weakens the impact of knowledge creation on service innovation, whereas low environmental uncertainty enhances this impact.

According to Fig 2, environmental uncertainty has different impacts on service innovation by moderating the relationship between knowledge creation and service innovation. In the case of high and low environmental uncertainty, the moderating effect of environmental uncertainty on the relationship between knowledge creation and service innovation presents different influencing mechanisms.

**(1) The influencing mechanism under high environmental uncertainty**

Under the high environmental uncertainty, the external environment is full of unstable factors, such as rapid technological progress, market demand fluctuations, policy changes, etc., which make service-oriented manufacturing enterprises face greater risks and uncertainties. In this environment, while digital platform capabilities can facilitate knowledge creation, the effectiveness and relevance of knowledge creation may be greatly affected. Due to the lack of stable market demand and forecast information, enterprises may encounter more difficulties in the process of knowledge creation, which in turn affects their ability to innovate. Specifically, knowledge creation may be too scattered or unclear, and it is difficult to form actual service innovation. ompanies’ R&D and innovation activities may become more conservative or delayed due to market uncertainty, leading to a slowdown or poor performance of service innovation. Therefore, under the high environmental uncertainty, the role of knowledge creation in promoting service innovation is weakened, and even if the capabilities of digital platforms are enhanced, it may be difficult for enterprises to effectively transform them into innovation results.

**(2) The influencing mechanism under low environmental uncertainty**

Under the low environmental uncertainty, the external environment is relatively stable, the market demand is relatively predictable, and the technology and policy changes are relatively slow. Such an environment is conducive to the strategic planning and resource allocation of enterprises, so that enterprises can carry out knowledge creation in a more targeted manner. Enterprises can rely on digital platform capabilities to effectively capture and analyze data, accumulate and innovate knowledge, and drive service innovation more smoothly. Specifically, knowledge creation can focus more on actual needs and market directions, and form innovative achievements with high application value. A stable market environment helps enterprises to gain more opportunities for trial and error in the innovation process, reduce risks, and accelerate the process of service innovation.

Therefore, under the low environmental uncertainty, the relationship between knowledge creation and service innovation is stronger, and enterprises can more smoothly transform knowledge creation into actual service innovation results. High environmental uncertainty reduces the role of knowledge creation in promoting service innovation, and enterprises may face more challenges in this environment, resulting in poor service innovation results. Low environmental uncertainty provides a more stable environment for innovation, and knowledge creation can more effectively drive service innovation. In the face of different degrees of environmental uncertainty, enterprises need to flexibly adjust their innovation strategies to maximize the advantages of digital platform capabilities and improve the effectiveness of service innovation.

## 5. Discussion

The findings of this study provide valuable insights into the mechanisms through which digital platform capabilities influence service innovation in service-oriented manufacturing enterprises. The results highlight the importance of digital platform capabilities, knowledge creation, and environmental uncertainty in shaping service innovation outcomes.

Digital platform capabilities play a crucial role in enhancing service innovation in service-oriented manufacturing enterprises. The study confirms that both digital platform integration and analysis capabilities and digital platform reconfiguration capabilities positively impact service innovation. These capabilities enable enterprises to effectively collect, analyze, and integrate data from various sources, thereby supporting the creation of innovative services. Additionally, the ability to dynamically reconfigure digital platforms allows enterprises to adapt quickly to changing market demands and customer needs, further driving service innovation. Our empirical analysis aligns with existing literature; for example, Boudreau (2010), from a producer’s perspective, found that enterprises’ digital platform capabilities allow them to integrate and share complementary assets and unique resources needed for innovation, thereby achieving collaborative innovation [68]. Roberts et al. (2022), from a consumer’s perspective, found that enterprises can better understand customer needs based on digital platforms, gain new market insights and innovative technological solutions, thus increasing the possibility of product innovation [69]. Turoń and Tóth (2023) critiqued the simplistic classification of shared mobility services as innovative without in-depth analysis, highlighting the need for detailed service analysis to avoid superficial innovation claims [70]. Building on existing research, our study further focuses on service-oriented manufacturing enterprises, divides digital platform capabilities into integration and analysis capabilities and reconfiguration capabilities, explores their impacts on service innovation by dimension, and emphasizes that enterprises need to flexibly utilize digital platform capabilities according to the degree of environmental uncertainty. Specifically, in highly uncertain environments, characterized by rapid technological changes and fluctuating market demands, enterprises must prioritize the flexibility and responsiveness of their digital platforms. In more stable environments, enterprises can focus on long-term service innovation and technology upgrading. Increasing investment in digital platforms and integrating advanced technologies, such as artificial intelligence and big data analytics, can significantly improve the intelligence level of the platform. The above research results are crucial for service-oriented manufacturing enterprises to leverage digital platform capabilities to achieve service innovation.

Knowledge creation emerges as a key mediator in the relationship between digital platform capabilities and service innovation. The study demonstrates that knowledge creation partially mediates the positive impact of digital platform integration and analysis capabilities on service innovation and the positive impact of digital platform reconfiguration capabilities on service innovation. This suggests that while digital platform capabilities provide the necessary infrastructure and tools, it is the process of knowledge creation that translates these capabilities into tangible service innovations. This finding is consistent with existing literature. Sun et al (2020) found that during the knowledge integration stage, enterprises not only use external resources for internal operations by integrating internal and external knowledge but also enhance the motivation to explore external resources [71]. Ode and Ayavoo (2020) revealed that various knowledge management activities have a significant positive impact on enterprise innovation performance [72]. Our study supplements existing research by showing that knowledge creation plays different roles in different dimensions of digital platform capabilities and acts on service innovation. Specifically, in terms of digital platform integration and analysis capabilities, knowledge creation provides favorable conditions for information processing, enabling enterprises to obtain the latest market information and improve their service models. In terms of digital platform reconfiguration capabilities, knowledge creation empowers both internal and external aspects of service-oriented manufacturing enterprises: it enhances service efficiency internally and improves the enterprise’s process reengineering capabilities externally. The research findings indicate that translating information obtained by enterprises through digital platforms into valuable new knowledge can promote service innovation, enhance enterprise competitiveness, and help enterprises escape the “servitization dilemma”.

Environmental uncertainty significantly influences the effectiveness of knowledge creation in driving service innovation. The study finds that environmental uncertainty negatively moderates the relationship between knowledge creation and service innovation. In highly uncertain environments, the challenges of rapid technological changes, fluctuating market demands, and unpredictable policy shifts can hinder the successful translation of knowledge creation into service innovation. Conversely, in more stable environments, enterprises have more opportunities to effectively utilize knowledge creation to drive service innovation. This finding aligns with existing research. Liao et al (2024) discovered that even with high-level digital platform capabilities, enterprises may fail to achieve high-quality innovative behaviors in highly uncertain external environments [73]. Highly uncertain environments exacerbate information asymmetry and market risks, weakening the prominent role of digital platform capabilities in information exchange and other aspects. This increases the difficulty of information processing and hinders enterprise innovation processes [74]. Building on existing studies, we further explored the boundary conditions of the relationship between knowledge creation and service innovation. The research emphasizes that enterprises need to adopt an agile management model to quickly adjust innovation strategies in turbulent environments. In stable environments, enterprises should adopt a balanced innovation approach, focusing on long-term strategic goals to ensure the sustainability and impact of innovation activities. This highlights the need for enterprises to develop strategies that enhance their adaptability and resilience in the face of environmental uncertainty.

## 6. Conclusion and implication

### 6.1. Conclusion

This paper elucidates the mechanism by which digital platform capability influences service innovation in service-oriented manufacturing enterprises. A questionnaire survey was designed based on domestic and international mature scales, with appropriate adjustments, and conducted both online and offline. Statistical analysis using SPSS and AMOS was performed on the research data. The results support all the hypotheses: digital platform capability positively promotes service innovation in service-oriented manufacturing enterprises; knowledge creation mediates the relationship between digital platform capability and service innovation; and environmental uncertainty negatively moderates the relationship between knowledge creation and service innovation.

### 6.2. Implication

#### 6.2.1. Theoretical implications.

Centered on the core context of digital transformation and service innovation in service-oriented manufacturing enterprises, this study advances the existing theoretical framework and expands the scope of relevant literature in a focused and in-depth manner across the following four dimensions. This is achieved through the integration of multi-theoretical perspectives and rigorous empirical validation, thereby yielding targeted and profound theoretical contributions:

Firstly, this study enriches research on the antecedents of service innovation and extends the literature boundaries in the service-oriented manufacturing field. Existing research on the antecedents of service innovation has predominantly focused on traditional manufacturing enterprises or pure service firms, with insufficient attention devoted to service-oriented manufacturing enterprises—entities defined by the in-depth integration of “manufacturing + service”. Furthermore, these studies rarely incorporate “digital platform capability”—a distinctive characteristic of the digital era—into their antecedent frameworks [21,61]. By leveraging the unique attributes of service-oriented manufacturing enterprises, namely “the embedding of services throughout the entire manufacturing process” and “customer participation in value co-creation” [3,4], this study empirically demonstrates the positive driving role of digital platform capabilities (encompassing digital platform integration and analysis capability, and digital platform reconstruction capability) in service innovation. Specifically, integration and analysis capability enables accurate alignment with market demands through data mining, while reconstruction capability facilitates responses to service iterations via flexible resource allocation. Collectively, these two capabilities form a dual-path mechanism characterized by “data-driven + dynamic adaptation”. This finding not only addresses the gap in the “digital capability dimension” within the antecedents of service innovation but also extends service innovation research from traditional contexts to the emerging domain of service-oriented manufacturing, thereby providing a benchmark framework for investigating the relationship between digital elements and innovation in this field.

Secondly, this study expands the research perspective on digital platform capability and opens the “capability-innovation” transformation black box. Prior literature on digital platform capability has primarily explored its overall impact on business model innovation and innovation performance from a macro perspective [75,76], or treated it merely as a moderating variable to examine contextual effects [77]. However, it has failed to decompose the micro-mechanisms underlying “how digital platform capability influences service innovation” [14,26]. This study fills this gap through two key breakthroughs: First, it decomposes digital platform capability into a two-dimensional construct consisting of “integration and analysis capability” and “reconstruction capability”, and verifies the heterogeneous contributions of these two dimensions to service innovation—overcoming the cognitive limitation of treating digital platform capability as a unidimensional concept. Second, it introduces knowledge creation as a mediating variable and validates the transformation path of “digital platform capability → knowledge spiral (socialization-externalization-combination-internalization) → service innovation” [52,54]. This mechanistic insight shifts research on digital platform capability from a “result-oriented” paradigm to a “process-oriented” one, thereby advancing the understanding of management science regarding the micro-level functioning of digital capabilities.

Thirdly, this study integrates institutional theory and the resource-based view to construct the chain logic of “institution-capability-innovation”. Previous studies have generally treated the institutional environment and firms’ internal capabilities as independent drivers of innovation: some have focused on the direct impact of institutions on innovation, while others have emphasized the core role of internal resources [34,43]. However, there remains a lack of research integrating the interactive relationship between these two factors [26]. This study is the first to empirically validate the chain path of “institutional environment →digital platform capability→knowledge creation→service innovation”. Specifically, the institutional environment does not exert a direct effect on innovation; instead, it influences innovation indirectly by “empowering firms’ internal digital capabilities”. For example, government subsidies for digitalization reduce the barriers to platform construction, enabling firms to acquire scarce digital resources, which are then converted into innovative outputs through knowledge creation. This finding not only provides contextualized empirical evidence for the cross-application of Institutional Theory and the Resource-Based View but also constructs a complete logical loop of “external institution - internal capability - intermediate process - innovation outcome”, thereby offering an integrated paradigm for innovation research from a cross-theoretical perspective.

Fourthly, this study clarifies the boundary constraints of environmental uncertainty and advances research on innovation boundary mechanisms. While existing studies have acknowledged the impact of environmental uncertainty on innovation, they have mostly conceptualized it as a “single external disturbance”, failing to distinguish between its different dimensions and rarely exploring its moderating effect on the “knowledge creation - service innovation” chain [59,73]. This study classifies environmental uncertainty into three distinct dimensions: “institutional uncertainty”, “market uncertainty”, and “technological uncertainty”, and empirically confirms that environmental uncertainty exerts a significant negative moderating effect on the relationship between knowledge creation and service innovation. Under conditions of high uncertainty, the time window for knowledge creation is compressed, and the costs of resource coordination increase—ultimately reducing the efficiency of converting knowledge into innovation [60,74]. This finding not only clarifies the “boundary constraint effect” of environmental uncertainty but also provides an analytical framework of “uncertainty type - innovation path matching” through dimensional decomposition, thereby enhancing the theoretical application of environmental factors in innovation research.

#### 6.2.2. Practical implications and recommendations.

Based on the study results, we put forward suggestions to promote the development of the Service-based manufacturing companies, which provide references for the government to formulate relevant policies and are of great practical significance.

Firstly, service-oriented manufacturing enterprises need to construct a “two-dimensional digital platform capability” dynamic empowerment system, systematically enhancing digital platform integration and analysis capabilities as well as digital platform reconfiguration capabilities through strategic planning. Specifically: First, enterprises should focus on the refined construction of digital platform integration and analysis capabilities, increasing investment in technologies such as artificial intelligence, big data analytics, and cloud computing to enhance the data processing and analysis capabilities of digital platforms. They should establish a cross-departmental data hub to achieve real-time integration of customer interaction data, supply chain data, and market dynamic data. Leveraging natural language processing and machine learning algorithms for demand forecasting, enterprises should also establish strategic partnerships with technology vendors to obtain advanced technical support and solutions. Second, enterprises need to increase the modular design of digital platform reconfiguration capabilities. They can adopt a microservice architecture to disassemble core functional modules, allowing quick invocation and recombination according to business needs, and design a flexible and scalable digital platform architecture to rapidly adapt to market changes and enterprise requirements. Meanwhile, developing standardized API interfaces, they should build a “service innovation alliance” with upstream and downstream partners, jointly test new service models, and shorten the iteration cycle of service solutions.

Secondly, service-oriented manufacturing enterprises need to transform information obtained through digital platforms into valuable new knowledge, creating an efficient “information-knowledge-innovation” conversion engine to drive service innovation. Specifically: First, enterprises should construct a knowledge management system, build a semantically linked “service knowledge map” based on digital platform data, perform labeling processing on unstructured information, form a reusable solution knowledge base, and promote knowledge collection, storage, sharing, and application. Second, cross-departmental collaboration should be encouraged to promote communication and cooperation among different departments, break down information silos, and accelerate knowledge flow. “Innovation sandbox” workshops should be established, and multi-department joint seminars organized, requiring each department to submit service innovation proposals based on digital platform data. Finally, employee training should be strengthened, and a knowledge conversion assessment mechanism established. The conversion rate of digital platform information should be incorporated into departmental KPIs, with assessment standards set. Meanwhile, certification training should be carried out to enhance employees’ knowledge conversion and innovation capabilities, creating new value for the enterprise.

Thirdly, service-oriented manufacturing enterprises should design a dynamic adaptation framework of “environmental resilience-innovation strategy”, reduce the impact of environmental uncertainty on service innovation through a three-level response mechanism, and adopt proactive adaptation strategies to mitigate its negative effects on service innovation. Specifically: First, establish an external environment monitoring system to track market dynamics, technological development trends, and policy changes in real time. Through data analysis and forecasting, potential risks and opportunities are identified in advance to provide a basis for enterprise decision-making.

Second, adopt agile management methods to enhance the enterprise’s adaptability and innovation capabilities. Build a corporate culture that encourages innovation and fosters a relaxed innovative atmosphere, enabling employees to dare to try new ideas and approaches. Finally, flexibly adjust resource allocation according to environmental changes to ensure the effective utilization of enterprise resources. In highly uncertain environments, enterprises can integrate third-party data services with internal platform data to construct a “market uncertainty index”, which automatically triggers the “innovation team” mechanism when the index exceeds a threshold. In stable environments, long-term technological service innovation can be invested in, and long-term cooperation platforms established with core customers and scientific research institutions to enhance the overall quality and speed of service innovation.

### 6.3. Limitations and future research

This paper has some limitations. Firstly, the research sample is limited to service-oriented manufacturing enterprises in the Jiangsu and Zhejiang regions, creating a regional selection bias. Future research should expand the sample to include service-oriented manufacturing enterprises from other regions to enhance the generalizability of the findings. Secondly, while this study uses questionnaire surveys and empirical analysis to construct theoretical models, these methods lack persuasive power compared to panoramic research paradigms. Future studies should consider combining methods such as simulation and experimental research to explore variable relationships in greater depth and enhance research logic. Finally, this article treats environmental uncertainty as a moderating variable, but environmental uncertainty is a multifaceted concept encompassing factors like market uncertainty and competitive intensity uncertainty. Future research should refine the concept of environmental uncertainty and explore its boundary mechanisms between digital platform capabilities, knowledge creation, and service innovation.

## Supporting information

S1 Data Data. (XLSX)
